# Thyroid Autoimmunity in Polycystic Ovary Syndrome: Phenotype Distribution, HDL-Cholesterol, and Data-Driven Clusters in a Retrospective Cohort Study

**DOI:** 10.3390/medicina62061184

**Published:** 2026-06-18

**Authors:** Raluca-Anamaria Mogoș, Alexandru Carauleanu, Ingrid-Andrada Vasilache, Simona Juliette Mogoș, Maria-Christina Ungureanu, Letitia Leustean, Iustina-Petra Condriuc, Sandra-Teodora Gavril, Ecaterina Tomaziu-Todosia Anton, Cristina Preda

**Affiliations:** 1 Grigore T. Popa University of Medicine and Pharmacy, 700115 Iasi, Romania; 2 Faculty of Medicine and Biological Sciences, ‘Ștefan cel Mare’ University, 720229 Suceava, Romania

**Keywords:** polycystic ovary syndrome, autoimmune thyroiditis, lipid profile, Polyendocrine metabolic ovarian syndrome

## Abstract

*Background and Objectives*: Autoimmune thyroiditis (AIT) is often reported patients with PCOS, and may co-occur with altered metabolic risk markers. The aims of this study were to assess baseline differences according to thyroid autoimmunity status, evaluate adjusted associations between thyroid autoimmunity and metabolic parameters, examine associations with PCOS phenotype distribution, and perform k-means clustering to explore data-driven subgroups and their autoimmune enrichment. *Materials and Methods*: We performed a retrospective cohort study of 651 women with PCOS, comparing those without AIT (n = 506) versus with AIT (n = 145). Associations between AIT and continuous outcomes (HDL; composite metabolic score) were evaluated using robust linear regression with HC3 standard errors and age modeled with a natural cubic spline (3 knots). The association between AIT and phenotype A was assessed via logistic regression with exponentiated coefficients (odds ratios). Unsupervised phenotyping used k-means clustering with silhouette analysis across k = 2…6. *Results*: Patients with AIT were older (median 40 vs. 35 years; *p* = 0.021). Phenotype distribution differed by AIT status (overall *p* = 0.029), with phenotype A less frequent among AIT-positive women (27% vs. 40%). In adjusted robust regression, AIT was associated with lower HDL by β = −4.34 mg/dL (95% CI −9.18 to 0.51; *p* = 0.081), while obesity (−7.04 mg/dL; *p* < 0.001) and diabetes (−6.47 mg/dL; *p* = 0.004) were associated with lower HDL. AIT was not associated with the composite metabolic score (β = −0.005; 95% CI −1.22 to 1.21; *p* = 0.994), whereas obesity was associated with higher score (β = 1.76; *p* = 0.003) and urban residence with lower score (β = −0.94; *p* = 0.011). In logistic regression, AIT was associated with lower odds of phenotype A (OR 0.63; 95% CI 0.41–0.97; *p* = 0.038), and hypertension was associated with higher odds of phenotype A (OR 1.91; 95% CI 1.20–3.04; *p* = 0.006). Silhouette analysis supported k=3 clusters (silhouette 0.349), and AIT prevalence was highest in cluster 3 (26.4%) versus clusters 1 (19.9%) and 2 (18.3%). *Conclusions*: AIT was associated with lower odds of phenotype A, and showed a borderline association with lower HDL-cholesterol but not with a composite metabolic score. Data-driven clustering identified a subgroup with higher autoimmune burden.

## 1. Introduction

Polycystic ovary syndrome is a common endocrine disorder and is frequently accompanied by insulin resistance and long-term metabolic risks such as type 2 diabetes mellitus, dyslipidemia, arterial hypertension, and endothelial dysfunction [1]. These factors contribute to a higher incidence of cardiovascular disease in women with PCOS across the life course. Alongside this metabolic risk profile, an expanding literature has emphasized the co-occurrence of autoimmune thyroid disease, particularly Hashimoto thyroiditis and thyroid autoantibody positivity, in women with PCOS, though the clinical meaning of this overlap remains debated and sometimes contradictory [2,3,4].

Multiple case–control studies report higher thyroid autoantibody positivity in PCOS than controls, including higher rates of anti-thyroid peroxidase (anti-TPO) and anti-thyroglobulin (anti-TG) antibodies and a higher prevalence of Hashimoto thyroiditis markers in PCOS cohorts. For example, Hepşen et al. evaluated thyroid autoimmunity in 184 euthyroid patients with polycystic ovary syndrome (PCOS) and 106 age-matched healthy controls, demonstrating significantly higher anti-thyroid peroxidase (anti-TPO) and anti-thyroglobulin (anti-TG) antibody levels in the PCOS group, despite the absence of significant differences in TSH and free thyroxine levels between groups. Moreover, the prevalence of anti-TPO and anti-TG antibody positivity was significantly increased among patients with PCOS, suggesting that thyroid autoimmunity may be more frequent in this population even in the absence of overt thyroid dysfunction [5]. Larger case–control evidence also supports increased anti-TPO levels in PCOS and suggests that routine screening for thyroid autoimmunity may be justified even when thyroid-stimulating hormone is normal [6].

A systematic review and meta-analysis found autoimmune thyroid disease in 26.03% of PCOS patients versus 9.72% in controls and reported a significant association between PCOS and autoimmune thyroid disease (OR 3.27, 95% CI 2.32–4.63), with persistent elevation across geographic subgroups [2]. This meta-analytic background has been used to argue that screening for thyroid function and thyroid-specific autoantibodies should be considered even in the absence of overt symptoms. Complementing prevalence-focused work, a cohort study using administrative data reported that autoimmune thyroid disease is associated with a higher risk of developing PCOS (adjusted hazard ratio 1.39; 95% CI 1.07–1.71) and that women with both autoimmune thyroid disease and PCOS have higher odds of diabetes, hyperlipidemia, and coronary artery disease, raising the possibility that autoimmunity may identify a PCOS subgroup with heightened cardiometabolic vulnerability [1].

However, the metabolic footprint of thyroid autoimmunity within PCOS is not uniform across studies. Some datasets suggest that Hashimoto thyroiditis in PCOS is associated with higher insulin secretion and insulin resistance indices and with a more adverse lipid profile, including higher total cholesterol and altered thyroid hormones in affected women [7,8]. In contrast, another study reported more limited metabolic differences between hypothyroid and euthyroid women with PCOS [9]. Although patients with PCOS demonstrated a higher prevalence of hypothyroidism and elevated anti-thyroid peroxidase antibody levels compared with healthy controls, hypothyroid PCOS patients showed significantly higher HDL-cholesterol levels, while no significant differences were identified for insulin resistance or most other metabolic parameters. Furthermore, thyroid autoimmunity was not associated with an increased risk of metabolic complications in that cohort [9]. In euthyroid PCOS specifically, thyroid autoantibody positivity has been associated with higher total cholesterol, LDL-cholesterol, and triglycerides, and authors have suggested that cardiovascular risk may be higher in euthyroid PCOS women with elevated thyroid autoantibodies than in those without them [10].

In parallel, PCOS itself is clinically heterogeneous. Rotterdam phenotypes A–D partition PCOS based on the combinations of hyperandrogenism, ovulatory dysfunction, and polycystic ovarian morphology. Phenotype-specific relationships between thyroid autoimmunity and PCOS have been investigated with mixed results: some studies report higher thyroid antibody positivity in grouped phenotypes A–B than in C–D and suggest phenotype-linked immune differences, whereas other studies do not find significant differences across phenotypes A–C relative to controls and report very low antibody positivity in phenotype D [11,12]. Additionally, a retrospective fertility-clinic cohort reported that PCOS patients with autoimmune thyroiditis may have less severe hyperandrogenemia (including lower testosterone and less frequent hirsutism) while also being more obese and possibly at elevated metabolic risk, illustrating the complexity of reproductive-metabolic tradeoffs in the PCOS–autoimmunity intersection [13].

Despite a rapidly growing literature base, there remains a need for analyses that concurrently (1) situate thyroid autoimmunity within PCOS phenotype distributions, (2) evaluate key cardiometabolic markers such as HDL-cholesterol while accounting for obesity and diabetes, and (3) explore whether unsupervised clustering can identify PCOS subgroups enriched for thyroid autoimmunity and autoimmune burden. The last point is motivated by the observation that autoimmune thyroid disease may coexist with other autoimmune conditions and that comorbidity patterns may reflect distinct pathophysiologic subtypes rather than simple additive risk [1].

Accordingly, in this retrospective cohort study of women with PCOS, we assessed baseline differences according to thyroid autoimmunity status, evaluated adjusted associations between thyroid autoimmunity and metabolic parameters, including lipid profile measures and a composite metabolic score, examined associations with PCOS phenotype distribution, and performed k-means clustering to explore data-driven subgroups and their autoimmune enrichment.

## 2. Materials and Methods

We conducted a retrospective cohort analysis of women with PCOS, comparing participants with and without thyroid autoimmunity. The cohort comprised 651 women with PCOS, including 506 without thyroid autoimmunity and 145 with thyroid autoimmunity.

Autoimmune thyroiditis was defined based on documented clinical diagnosis or laboratory evidence of thyroid autoimmunity, and was coded as a binary variable (present vs. absent). Because of the retrospective study design, no a priori sample size calculation was performed. The study included all consecutive eligible patients identified in the institutional database during the predefined study period. A post hoc power analysis based on the final analytical cohort (506 women with PCOS without autoimmune thyroiditis and 145 women with PCOS and autoimmune thyroiditis) demonstrated that the available sample size provided approximately 80% statistical power to detect small between-group effect sizes (Cohen’s d = 0.26) at a two-sided significance level of 0.05.

PCOS phenotypes were classified according to the Rotterdam criteria into four categories (A–D), based on the presence of hyperandrogenism, ovulatory dysfunction, and polycystic ovarian morphology. Phenotype A was used as the reference category in regression analyses.

Clinical covariates included age (modeled as a continuous variable), obesity, diabetes mellitus, hypertension, smoking status (excluded from final models), and residential setting (urban vs. rural). Obesity, diabetes, and hypertension were treated as binary variables based on documented diagnoses.

Lipid profile parameters included total cholesterol, LDL-cholesterol, and HDL-cholesterol. Hormonal parameters included serum testosterone, dehydroepiandrosterone sulfate (DHEAS), thyroid-stimulating hormone (TSH), free thyroxine (FT4), and the luteinizing hormone to follicle-stimulating hormone ratio (LH/FSH). In order to improve normality, skewed variables (testosterone, DHEAS, and TSH) were log-transformed prior to regression analyses.

Other covariates included clinical hyperandrogenism signs, biochemical hyperandrogenism, menstrual cycle disorders, polycystic ovarian morphology on ultrasound, PCOS phenotype categories (A–D), and medication use (metformin, levothyroxine).

Baseline differences were summarized using medians (Q1, Q3) for continuous variables and counts (percentages) for categorical variables. Group comparisons used Wilcoxon rank-sum tests and Fisher’s exact tests as specified in the dataset output.

Multivariable linear regression models were used to assess the association between AIT and lipid parameters (HDL-cholesterol, LDL-cholesterol, and total cholesterol). Age was modeled using natural cubic splines with three degrees of freedom to account for potential non-linear effects. All models were adjusted for obesity, diabetes mellitus, hypertension, and residential setting.

Separate multivariable linear regression models were constructed for each hormonal outcome (log-transformed testosterone, log-transformed DHEAS, LH/FSH ratio, log-transformed TSH, and FT4), with AIT as the main exposure and the same set of covariates as above.

Additional exploratory analyses were performed in subsets of patients with available data to further evaluate the potential influence of obesity, thyroid-related parameters, and treatment exposure on the observed metabolic associations. Exploratory descriptive analyses comparing BMI, thyroid antibody titers, thyroid functional status, and treatment exposure according to autoimmune thyroiditis status were summarized in Supplementary Table S1. Additionally, supplementary multivariable sensitivity analyses were performed to evaluate whether the associations between autoimmune thyroiditis and lipid parameters remained stable after adjustment for BMI, diabetes mellitus, hypertension, and treatment exposure variables. Separate linear regression models were constructed for total cholesterol, HDL-cholesterol, and LDL-cholesterol levels. Age was modeled using restricted cubic splines with 3 degrees of freedom to account for potential non-linear effects. These supplementary analyses are presented in Supplementary Table S2. Because these variables were not systematically available for the entire cohort due to the retrospective design, all supplementary analyses were considered exploratory and were interpreted cautiously.

The association between PCOS phenotype and lipid or hormonal parameters was evaluated using multivariable linear regression models, with phenotype A as the reference category. Models were adjusted for AIT status and clinical covariates.

In order to investigate whether the effect of AIT differed across PCOS phenotypes, interaction terms between AIT and phenotype were included in regression models. The statistical significance of interaction terms was used to assess effect modification.

The association between AIT and PCOS phenotype distribution was initially evaluated using the chi-square test. Subsequently, multinomial logistic regression models were used to estimate adjusted odds ratios (ORs) and 95% confidence intervals (CIs) for each phenotype relative to phenotype A. A two-sided *p*-value < 0.05 was considered statistically significant. All analyses were performed using R statistical software (version 4.5.3, R Foundation for Statistical Computing, Vienna, Austria).

For unsupervised clustering, we applied k-means clustering and selected the number of clusters based on silhouette analysis across k = 2…6, reporting average silhouette width for each k.

## 3. Results

Table 1 summarizes baseline characteristics by thyroid autoimmunity status. Patients with autoimmune thyroiditis were significantly older compared to those without autoimmune thyroiditis (median age 40 [28–46] vs. 35 [24–45] years, *p* = 0.021). Metabolic comorbidities, including obesity, diabetes mellitus, and hypertension, were similar between groups. Markers of androgen excess and reproductive dysfunction showed no statistically significant differences between groups.

On the other hand, the distribution of PCOS phenotypes differed significantly according to autoimmune thyroiditis status (*p* = 0.029). Phenotype A was less frequent among patients with autoimmune thyroiditis (27% vs. 40%), whereas phenotype B was more prevalent (37% vs. 27%). The distribution of phenotypes C and D was comparable between groups.

Regarding treatment, metformin use tended to be lower in patients with autoimmune thyroiditis (2.8% vs. 7.1%), although this did not reach statistical significance (*p* = 0.075). As expected, levothyroxine use was significantly higher in the autoimmune thyroiditis group (25% vs. 15%, *p* = 0.009).

In multivariable regression models adjusted for age (modeled using natural splines), obesity, diabetes mellitus, hypertension, and residential setting, autoimmune thyroiditis was not significantly associated with lipid parameters in patients with PCOS (Table 2). A borderline trend toward lower HDL-cholesterol levels was observed in patients with autoimmune thyroiditis (β = −4.44 mg/dL, *p* = 0.071), while no significant associations were identified for total cholesterol (β = −6.10 mg/dL, *p* = 0.284) or LDL-cholesterol (β = 5.17 mg/dL, *p* = 0.442).

Age showed a strong non-linear relationship with both total cholesterol and LDL-cholesterol levels, with higher spline terms being significantly associated with increased lipid values. In contrast, obesity (β = −7.13 mg/dL, *p* < 0.001) and diabetes mellitus (β = −6.46 mg/dL, *p* = 0.004) were independently associated with significantly lower HDL-cholesterol levels. No significant associations were observed for hypertension or urban residence across the lipid outcomes.

Additional exploratory analyses including BMI, thyroid antibody titers, thyroid functional status, and treatment exposure were performed in subsets of patients with available data (Supplementary Tables S1 and S2). BMI did not differ significantly between PCOS patients with and without autoimmune thyroiditis. After adjustment for BMI, diabetes mellitus, hypertension, and treatment exposure variables, autoimmune thyroiditis remained not significantly associated with total cholesterol, HDL-cholesterol, or LDL-cholesterol levels in multivariable sensitivity analyses.

**Figure 1:**
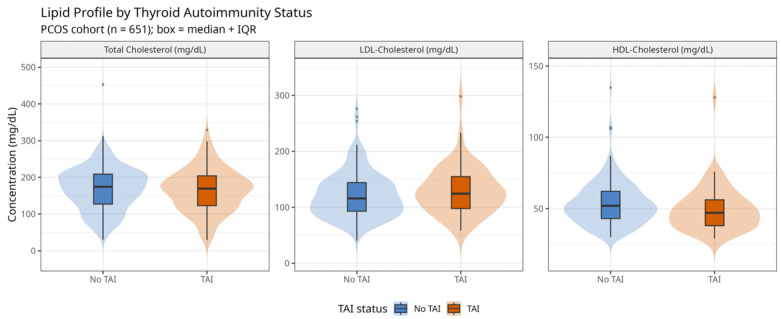
Violin plots representing the distribution of lipid profile parameters by thyroid autoimmunity status.

Figure 1 displays the unadjusted distribution of HDL, LDL and total cholesterol by AIT status. Visual inspection of the lipid profile distributions showed largely overlapping patterns between patients with and without autoimmune thyroiditis. Total cholesterol levels were similar between groups (median 174 vs. 169 mg/dL, *p* = 0.5), while LDL-cholesterol levels were slightly higher in the autoimmune thyroiditis group (125 vs. 116 mg/dL), although this difference did not reach statistical significance (*p* = 0.2).

The HDL-cholesterol levels were lower in patients with autoimmune thyroiditis (47 vs. 52 mg/dL), and this difference was statistically significant in unadjusted analyses (*p* = 0.042). However, this association was attenuated after multivariable adjustment, suggesting that the observed difference may be partly explained by underlying clinical factors rather than thyroid autoimmunity itself.

In the descriptive analysis, lipid profiles showed limited variation across PCOS phenotypes and AIT status (Table 3). Among women without AIT, median total cholesterol ranged from 159 mg/dL in phenotype A to 199 mg/dL in phenotype B. LDL-cholesterol was highest in phenotype C (134.5 mg/dL), while HDL-cholesterol values were relatively similar across phenotypes (49–53 mg/dL). Differences across phenotypes were statistically significant only for total cholesterol (*p* = 0.001), but not for LDL (*p* = 0.502) or HDL (*p* = 0.606).

Among women with AIT, lipid levels were comparable across phenotypes, with median total cholesterol ranging from 165.5 to 174 mg/dL, LDL from 108 to 135 mg/dL, and HDL from 39 to 51 mg/dL. No statistically significant differences were observed across phenotypes for any lipid parameter (all *p* > 0.2).

In adjusted stratified regression analyses, among women without AIT, phenotype C was associated with higher total cholesterol compared to phenotype A (β = 17.46 mg/dL, 95% CI: 2.49–32.43, *p* = 0.022), while phenotypes B and D were not significantly associated. No significant associations were found between PCOS phenotype and LDL- or HDL-cholesterol (Table 4).

Among women with AIT, no significant associations were observed between PCOS phenotype and any lipid parameter, although a non-significant trend toward lower HDL-cholesterol was noted in phenotype D (β = −8.76 mg/dL, *p* = 0.101).

In the overall model including interaction terms, phenotype C remained significantly associated with higher total cholesterol (β = 16.79 mg/dL, 95% CI: 2.00–31.57, *p* = 0.026). However, no significant interactions between PCOS phenotype and AIT were identified for any lipid outcome (all *p* > 0.05), suggesting that the relationship between phenotype and lipid profile does not differ according to AIT status.

**Table 4:** Adjusted associations between PCOS phenotype and lipid profile including interaction with autoimmune thyroiditis.

Outcome	Term	β (95% CI)	*p*-Value
Total cholesterol (mg/dL)	Phenotype B	9.75 (−4.35 to 23.85)	0.175
	Phenotype C	16.79 (2.00 to 31.57)	0.026
	Phenotype D	8.62 (−5.44 to 22.69)	0.229
	AIT	1.99 (−16.51 to 20.48)	0.833
	Phenotype B × AIT	−11.18 (−37.38 to 15.02)	0.402
	Phenotype C × AIT	−17.74 (−50.31 to 14.83)	0.285
	Phenotype D × AIT	−11.39 (−47.55 to 24.77)	0.536
LDL-cholesterol (mg/dL)	Phenotype B	0.17 (−18.50 to 18.85)	0.985
	Phenotype C	8.59 (−6.63 to 23.82)	0.267
	Phenotype D	2.51 (−17.30 to 22.32)	0.803
	AIT	7.12 (−14.41 to 28.65)	0.515
	Phenotype B × AIT	−2.17 (−35.79 to 31.45)	0.899
	Phenotype C × AIT	−5.51 (−38.82 to 27.81)	0.745
	Phenotype D × AIT	−3.35 (−48.49 to 41.79)	0.884
HDL-cholesterol (mg/dL)	Phenotype B	0.31 (−6.11 to 6.73)	0.924
	Phenotype C	−3.84 (−9.85 to 2.17)	0.209
	Phenotype D	−0.06 (−6.31 to 6.20)	0.986
	AIT	−1.75 (−10.07 to 6.57)	0.678
	Phenotype B × AIT	−4.05 (−17.74 to 9.64)	0.560
	Phenotype C × AIT	1.81 (−10.30 to 13.92)	0.769
	Phenotype D × AIT	−6.36 (−17.19 to 4.48)	0.249

Legend: LDL = low-density lipoprotein cholesterol; HDL = high-density lipoprotein cholesterol; AIT = autoimmune thyroiditis; CI = confidence interval.

In multivariable-adjusted analyses, AIT was not significantly associated with markers of androgen excess in patients with PCOS (Table 5). Specifically, no independent associations were observed between AIT and serum testosterone or DHEAS levels. Similarly, free thyroxine (FT4) levels were not significantly different according to AIT status. However, AIT was strongly associated with higher TSH levels (β = 0.73 on the log scale, *p* < 0.001), confirming the expected impact of thyroid autoimmunity on the hypothalamic–pituitary–thyroid axis. A borderline inverse association was observed for the LH/FSH ratio (β = −0.36, *p* = 0.085), suggesting a potential, but non-significant, influence of AIT on gonadotropin dynamics.

When examining the association between PCOS phenotype and hormonal parameters, phenotype B and especially phenotype D were associated with significantly lower testosterone levels compared with phenotype A, independent of AIT and other covariates (Table 6). No significant differences were observed for phenotype C. These findings suggest that the hyperandrogenic phenotype (A) retains the highest androgen levels, while non-classic phenotypes may exhibit a milder biochemical androgen profile.

Interaction analyses showed no significant effect modification by AIT on the relationship between PCOS phenotype and hormonal parameters (Table 7). The absence of significant interaction terms across all models indicates that thyroid autoimmunity does not meaningfully alter the hormonal expression of different PCOS phenotypes.

In multinomial logistic regression models, AIT was associated with increased odds of phenotype B compared with phenotype A (OR 1.76, *p* = 0.028), while no significant associations were observed for phenotypes C or D (Table 8). This suggests that AIT may be linked to a shift in phenotype distribution, favoring certain non-classic PCOS phenotypes, although this association appears modest and not consistent across all phenotype categories.

Silhouette analysis was performed to evaluate the optimal number of clusters in the k-means solutions (Table 9). The highest average silhouette width was observed for the three-cluster solution (k = 3; silhouette = 0.349), indicating the best overall balance between within-cluster cohesion and between-cluster separation.

Two-cluster (k = 2) and six-cluster (k = 6) solutions also showed relatively similar silhouette values (0.337 and 0.331, respectively), suggesting moderate clustering structure. On the other hand, solutions with four and five clusters demonstrated lower silhouette values (0.307 and 0.305), indicating poorer separation and less well-defined clusters. Overall, the silhouette coefficients suggested moderate clustering performance, indicating that the identified clusters should be interpreted as exploratory patterns rather than clearly distinct clinical entities.

The prevalence of autoimmune thyroiditis varied across clusters, ranging from 18.3% to 26.4% (Table 10). Cluster 3 showed the highest prevalence of autoimmune thyroiditis (26.4%), compared to cluster 1 (19.9%) and cluster 2 (18.3%). Despite this variation, the differences in TAI prevalence across clusters were relatively modest, suggesting that autoimmune thyroiditis is not strongly associated with cluster membership and the overall between-cluster differences did not reach statistical significance (*p* = 0.072).

The distribution of autoimmune status differed slightly across clusters of PCOS patients (Table 11). Cluster 3 included the highest number of patients with autoimmune thyroiditis, both as an isolated condition (n = 20) and in combination with other autoimmune diseases (n = 77), compared to clusters 1 and 2. However, clusters 1 and 2 were characterized by a higher proportion of patients without autoimmune disease. Isolated autoimmune thyroiditis was relatively uncommon across all clusters, with low absolute counts in each group. The overall between-cluster differences did not reach statistical significance (*p* = 0.083), supporting the exploratory nature of the identified clustering patterns.

## 4. Discussion

This retrospective cohort analysis adds three main observations to the PCOS–thyroid autoimmunity literature. First, thyroid autoimmunity was associated with different Rotterdam phenotype distributions and lower odds of phenotype A. Second, thyroid autoimmunity showed a borderline association with lower HDL-cholesterol after adjustment but no association with a composite metabolic score. Third, unsupervised clustering identified a subgroup with higher prevalence of thyroid autoimmunity and a higher burden of concomitant autoimmunity.

The finding that phenotype distribution differed by thyroid autoimmunity status is consistent with prior work suggesting that thyroid autoantibodies may vary across phenotype groupings, with some studies finding higher thyroid antibody positivity in phenotypes A–B compared with C–D [11]. At the same time, the literature is not uniform. Another phenotype-focused analysis reported no differences in TPOAb positivity across phenotypes A–C relative to controls (and noted very low positivity in phenotype D), emphasizing that phenotype–autoimmunity relationships may be context-dependent, potentially influenced by population, treatment, and ascertainment factors [12]. The present cohort’s association between thyroid autoimmunity and lower odds of phenotype A (OR 0.63; 95% CI 0.41–0.97) also aligns directionally with retrospective cohort data suggesting that PCOS patients with autoimmune thyroiditis may show less severe hyperandrogenemia and hyperandrogenism, including lower testosterone and less frequent hirsutism [13].

The association between thyroid autoimmunity and HDL in PCOS is particularly complex in the literature. In the current adjusted model, thyroid autoimmunity was associated with lower HDL by 4.34 mg/dL, but this did not reach conventional statistical significance (*p* = 0.081). Prior studies in PCOS have reported divergent HDL patterns depending on thyroid functional status and cohort characteristics. One study observed substantially higher HDL in hypothyroid PCOS patients than in non-hypothyroid PCOS patients while finding no difference in insulin resistance, and concluded that autoimmunity was not linked to a higher risk of metabolic problems in that sample [9]. In contrast, other studies examining women with combined Hashimoto thyroiditis and PCOS reported lower HDL and markedly adverse lipid profiles compared with controls, indicating that when thyroid disease and PCOS co-occur, dyslipidemia may be pronounced [7,14].

Our finding that obesity and diabetes were strongly associated with lower HDL is consistent with the broader recognition that PCOS is characterized by insulin resistance and long-term metabolic risks, including dyslipidemia and type 2 diabetes mellitus, which are key determinants of cardiovascular risk in PCOS populations [15,16]. The lack of association between thyroid autoimmunity and the composite metabolic score in our data may reflect the heterogeneity of metabolic manifestations across PCOS and thyroid autoimmunity subtypes, a point echoed in the literature describing controversial and inconsistent findings on the PCOS–autoimmunity relationship [17,18]. It is also consistent with evidence that certain metabolic abnormalities may emerge more clearly when thyroid dysfunction (such as subclinical hypothyroidism) is present, rather than autoantibody positivity alone [19,20]. In one study, subclinical hypothyroid PCOS participants had higher HOMA-IR and more dyslipidemia than euthyroid PCOS and controls [21].

Although our primary metabolic models focused on HDL and a composite score, prior PCOS literature provides plausible reasons to examine insulin resistance pathways in future analyses. Several studies report higher insulin resistance indices or fasting insulin in PCOS women with autoimmune thyroiditis compared with those without thyroid autoimmunity, and authors have concluded that Hashimoto thyroiditis in PCOS may relate to insulin resistance and relatively lower thyroid function [22,23]. Conversely, some studies report that insulin resistance does not differ between hypothyroid and non-hypothyroid PCOS patients, an aspect which highlights the need for careful stratification by thyroid function, antibody titers, and treatment status [24,25].

In additional exploratory analyses, adjustment for BMI, cardiometabolic comorbidities, and treatment exposure did not materially alter the observed associations between autoimmune thyroiditis and lipid parameters. Specifically, autoimmune thyroiditis remained not significantly associated with total cholesterol, HDL-cholesterol, or LDL-cholesterol levels after multivariable adjustment. Although BMI demonstrated a borderline inverse association with HDL-cholesterol levels, no significant associations were observed between BMI and total cholesterol or LDL-cholesterol levels. Furthermore, treatment-related variables, including levothyroxine therapy, metformin use, and oral contraceptive exposure, did not significantly modify the observed lipid profile associations. These findings suggest that the absence of major metabolic differences between PCOS patients with and without autoimmune thyroiditis in our cohort was not substantially explained by obesity or treatment-related confounding in the available subset of patients.

Our exploratory clustering analysis identified three clusters with modest silhouette separation. Cluster 3 showed the highest prevalence of thyroid autoimmunity and also the highest counts in the “AIT + other autoimmune” category, suggesting that a subgroup of PCOS patients may exhibit a broader autoimmune comorbidity pattern. This observation conceptually aligns with population-based evidence indicating that women with both autoimmune thyroid disease and PCOS have higher odds of diabetes, hyperlipidemia, and coronary artery disease than women without this comorbidity pairing, implying that immune-metabolic clustering could capture clinically meaningful risk strata [26]. Recent efforts to redefine the conceptual framework of polycystic ovary syndrome further support the importance of investigating multisystem interactions, including endocrine, metabolic, and immune mechanisms. In 2026, an international consensus initiative published in The Lancet proposed replacing the term polycystic ovary syndrome with Polyendocrine metabolic ovarian syndrome (PMOS), emphasizing that the traditional terminology insufficiently reflects the complex systemic nature of the disorder [26].

Importantly, because cohort studies may lack laboratory phenotyping (including insulin and thyroid function data), detailed clinical cohorts like the present dataset can complement such evidence by providing mechanistically adjacent biomarkers and phenotype labels for subgroup discovery.

Because autoimmune thyroid disease appears to be frequent in PCOS, and because some studies suggest lipid and cardiovascular risk implications even in euthyroid PCOS women with elevated thyroid autoantibodies, incorporating thyroid autoimmunity assessment into PCOS evaluation may be clinically useful, particularly in patients with additional metabolic risk factors or symptom evolution over time. Several sources explicitly recommend assessing thyroid autoantibodies in women with PCOS and considering screening even in the absence of overt thyroid symptoms [27,28,29]. In the context of our data, the phenotype shift and the cluster enrichment signal support the pragmatic view that thyroid autoimmunity status may contribute to refined PCOS phenotyping rather than acting solely as a binary metabolic risk amplifier.

This study’s strengths include a reasonably sized PCOS cohort with phenotype classification and the use of robust regression with flexible age adjustment, which may improve inference in the presence of heteroskedasticity and nonlinear age associations. The integration of unsupervised clustering with autoimmune burden characterization provides an exploratory framework for identifying subgroups.

Limitations include the retrospective design, potential heterogeneity in the operational definition of thyroid autoimmunity in clinical records, and the modest silhouette values indicating limited separation between clusters. Additionally, antibody titers (not only positivity) may matter for metabolic associations (e.g., very high anti-TPO titers have been linked to higher fasting insulin prevalence and lower HDL in some non-PCOS settings), suggesting that future PCOS studies should incorporate antibody titer gradients where available. Due to the retrospective study design, BMI, thyroid antibody titers, thyroid functional status, and treatment-related data were only available in subsets of patients, and therefore the corresponding analyses should be considered exploratory and interpreted cautiously. Also, important limitations of this retrospective study include the absence of systematically recorded HOMA-IR, duration of PCOS and detailed longitudinal treatment exposure. Thus, residual confounding related to these variables cannot be excluded and should be further evaluated. Finally, the temporal relationship between PCOS diagnosis and the onset or treatment initiation of thyroid disease could not be systematically established for all patients, and therefore potential diagnostic overlap in isolated cases cannot be completely excluded.

## 5. Conclusions

In euthyroid women with PCOS, autoimmune thyroiditis was not independently associated with adverse lipid profiles after adjustment for obesity, diabetes, and age, suggesting that metabolic alterations are driven primarily by metabolic comorbidities rather than thyroid autoimmunity itself. Obesity and diabetes emerged as the main determinants of reduced HDL-cholesterol, while age showed a strong non-linear association with total and LDL cholesterol levels.

AIT was associated with higher TSH levels and a shift toward phenotype B relative to phenotype A, supporting a potential link between thyroid autoimmunity and a milder androgenic PCOS phenotype. Phenotypes B and D were independently associated with lower testosterone levels regardless of AIT status.

Cluster analysis identified heterogeneous PCOS subgroups with modest differences in AIT prevalence, suggesting that thyroid autoimmunity may cluster within specific clinical profiles.

Given meta-analytic evidence that autoimmune thyroid disease is more common in PCOS than in controls and published recommendations that thyroid function and autoantibody screening should be considered in women with PCOS, our results support considering thyroid autoimmunity and thyroid functional assessment in the broader clinical evaluation of patients with PCOS and motivate prospective studies to determine whether autoimmunity-informed clusters predict longitudinal cardiometabolic outcomes and cardiovascular events.

## Figures and Tables

**Table 1:** Baseline characteristics of PCOS patients according to autoimmune thyroiditis status.

Characteristic	PCOS, No AIT (N = 506)	PCOS and AIT (N = 145)	*p*-Value
Age, years	35 (24, 45)	40 (28, 46)	0.021
• Rural residence	224 (44%)	63 (43%)	>0.9
• Urban residence	282 (56%)	82 (57%)	
Obesity	276 (55%)	89 (61%)	0.20
Diabetes mellitus	121 (24%)	32 (22%)	0.70
Hypertension	136 (27%)	29 (20%)	0.10
Smoking	5 (1.0%)	4 (2.8%)	0.12
Clinical hyperandrogenism	170 (34%)	37 (26%)	0.069
Biochemical hyperandrogenism	80 (16%)	18 (12%)	0.40
Menstrual dysfunction	196 (39%)	45 (31%)	0.10
Polycystic ovarian morphology	505 (100%)	143 (99%)	0.13
PCOS phenotype			
• Phenotype A	201 (40%)	39 (27%)	0.029
• Phenotype B	138 (27%)	53 (37%)	
• Phenotype C	75 (15%)	24 (17%)	
• Phenotype D	92 (18%)	29 (20%)	
Metformin use	36 (7.1%)	4 (2.8%)	0.075
Levothyroxine use	77 (15%)	36 (25%)	0.009

Legend: PCOS = polycystic ovary syndrome; AIT = autoimmune thyroiditis.

**Table 2:** Multivariable linear regression model for lipid parameters levels.

Term	HDL β (95% CI)	*p*	Total Cholesterol β (95% CI)	*p*	LDL β (95% CI)	*p*
Autoimmune thyroiditis	−4.44 (−9.26, 0.38)	0.071	−6.10 (−17.28, 5.08)	0.284	5.17 (−8.06, 18.40)	0.442
Age (spline 1)	6.57 (−1.63, 14.76)	0.116	47.40 (28.11, 66.69)	<0.001	24.37 (−0.55, 49.28)	0.055
Age (spline 2)	−1.02 (−24.33, 22.30)	0.932	122.20 (72.04, 172.36)	<0.001	69.26 (29.19, 109.33)	<0.001
Age (spline 3)	2.54 (−11.34, 16.41)	0.719	35.53 (5.72, 65.35)	0.020	24.56 (−9.24, 58.36)	0.153
Obesity	−7.13 (−11.18, −3.08)	<0.001	−6.36 (−15.92, 3.20)	0.192	7.91 (−4.75, 20.57)	0.220
Diabetes mellitus	−6.46 (−10.85, −2.06)	0.004	−7.64 (−19.55, 4.26)	0.208	−3.44 (−17.58, 10.70)	0.632
Hypertension	−1.14 (−6.71, 4.42)	0.686	0.24 (−11.49, 11.98)	0.968	1.22 (−11.34, 13.78)	0.849
Urban residence	2.64 (−1.24, 6.51)	0.181	−6.49 (−15.60, 2.62)	0.162	−3.19 (−14.36, 7.98)	0.574

Legend: HDL = high-density lipoprotein cholesterol; LDL = low-density lipoprotein cholesterol; CI = confidence interval.

**Table 3:** Adjusted associations between PCOS phenotype and lipid profile, stratified by thyroid autoimmunity.

Outcome	Phenotype (ref = A)	No AIT: β (95% CI)	*p*-Value	AIT: β (95% CI)	*p*-Value
Total cholesterol (mg/dL)	B	9.95 (−4.50 to 24.40)	0.177	1.89 (−23.62 to 27.39)	0.884
	C	17.46 (2.49 to 32.43)	0.022	−0.75 (−31.25 to 29.75)	0.961
	D	8.27 (−5.90 to 22.45)	0.252	1.81 (−31.60 to 35.22)	0.915
LDL-cholesterol (mg/dL)	B	−2.05 (−20.86 to 16.77)	0.830	2.59 (−33.27 to 38.46)	0.885
	C	7.79 (−7.63 to 23.21)	0.320	−2.79 (−43.42 to 37.84)	0.891
	D	1.40 (−18.43 to 21.23)	0.889	8.72 (−36.39 to 53.82)	0.699
HDL-cholesterol (mg/dL)	B	0.64 (−5.95 to 7.23)	0.848	−5.85 (−18.77 to 7.07)	0.367
	C	−3.47 (−9.49 to 2.54)	0.256	−4.95 (−23.95 to 14.05)	0.603
	D	−0.14 (−6.65 to 6.37)	0.966	−8.76 (−19.29 to 1.76)	0.101

Legend: HDL = high-density lipoprotein cholesterol; LDL = low-density lipoprotein cholesterol; AIT = autoimmune thyroiditis; CI = confidence interval.

**Table 5:** Adjusted associations between autoimmune thyroiditis and hormonal profile.

Outcome	β (95% CI)	*p*-Value
Log testosterone	−0.29 (−1.05 to 0.46)	0.445
Log DHEAS	0.30 (−0.38 to 0.97)	0.388
LH/FSH ratio	−0.36 (−0.77 to 0.05)	0.085
Log TSH	0.73 (0.40 to 1.05)	<0.001
FT4	11.26 (−10.18 to 32.69)	0.302

Legend: DHEAS = dehydroepiandrosterone sulfate; LH = luteinizing hormone; FSH = follicle-stimulating hormone; TSH = thyroid-stimulating hormone; FT4 = free thyroxine; CI = confidence interval.

**Table 6:** Adjusted associations between PCOS phenotype and testosterone (log-transformed).

Term	β (95% CI)	*p*-Value
Phenotype B	−1.63 (−3.18 to −0.07)	0.040
Phenotype C	−0.11 (−0.91 to 0.70)	0.798
Phenotype D	−2.12 (−2.97 to −1.26)	<0.001

Legend: CI = confidence interval.

**Table 7:** Interaction between PCOS phenotype and AIT for hormonal parameters.

A. Testosterone (log)		
**Term**	**β (95% CI)**	***p*** **-Value**
AIT	−0.29 (−1.26 to 0.68)	0.555
AIT × Phenotype B	0.05 (−2.25 to 2.36)	0.965
AIT × Phenotype C	0.65 (−1.45 to 2.75)	0.544
AIT × Phenotype D	0.20 (−1.51 to 1.90)	0.820
B. DHEAS (log)		
**Term**	**β (95% CI)**	***p*** **-Value**
AIT	0.28 (−0.66 to 1.23)	0.553
AIT × Phenotype B	0.16 (−1.13 to 1.45)	0.804
AIT × Phenotype C	0.25 (−1.31 to 1.81)	0.752
AIT × Phenotype D	−0.33 (−4.37 to 3.70)	0.870
C. LH/FSH ratio		
**Term**	**β (95% CI)**	***p*** **-Value**
AIT	−0.22 (−0.78 to 0.35)	0.449
AIT × Phenotype B	−0.10 (−1.34 to 1.14)	0.874
AIT × Phenotype C	0.15 (−1.44 to 1.75)	0.849
AIT × Phenotype D	−0.61 (−1.42 to 0.20)	0.139

Legend: AIT = autoimmune thyroiditis; DHEAS = dehydroepiandrosterone sulfate; LH = luteinizing hormone; FSH = follicle-stimulating hormone; CI = confidence interval.

**Table 8:** Multinomial logistic regression: association between AIT and PCOS phenotype.

Phenotype	OR (95% CI)	*p*-Value
B vs. A	1.76 (1.06 to 2.91)	0.028
C vs. A	1.60 (0.90 to 2.86)	0.112
D vs. A	1.50 (0.86 to 2.61)	0.151

Legend: OR = odds ratio; CI = confidence interval.

**Table 9:** Silhouette analysis for k-means clustering solutions.

Number of Clusters (k)	Average Silhouette Width
2	0.337
3	0.349
4	0.307
5	0.305
6	0.331

**Table 10:** Cluster distribution by thyroid autoimmunity status.

Cluster	No AIT	AIT	Total	AIT Prevalence	*p* Value
1	121	30	151	19.9%	0.072
2	170	38	208	18.3%	
3	215	77	292	26.4%	

Legend: AIT = autoimmune thyroiditis.

**Table 11:** Cluster distribution by autoimmune burden.

Cluster	No Autoimmunity	Isolated AIT	AIT + Other Autoimmune	Total	*p* Value
1	112 (74.2%)	9 (6.0%)	30 (19.9%)	151	0.083
2	162 (77.9%)	8 (3.8%)	38 (18.3%)	208	
3	195 (66.8%)	20 (6.8%)	77 (26.4%)	292	

Legend: AIT = autoimmune thyroiditis.

## Data Availability

The datasets are available from the corresponding authors upon reasonable request due to local policies.

## Supplementary Materials

The following supporting information can be downloaded at https://www.mdpi.com/article/10.3390/medicina62061184/s1: Table S1. BMI, thyroid-related parameters, and treatment characteristics according to autoimmune thyroiditis status in women with PCOS. Table S2. Multivariable sensitivity analyses evaluating the association between autoimmune thyroiditis and lipid parameters after adjustment for BMI, cardiometabolic comorbidities, and treatment exposure.

## Abbreviations

The following abbreviations are used in this manuscript:

AIT	Autoimmune thyroiditis
anti-TG	Anti-thyroglobulin antibodies
anti-TPO	Anti-thyroid peroxidase antibodies
BMI	Body mass index
CI	Confidence interval
DHEAS	Dehydroepiandrosterone sulfate
FT4	Free thyroxine
HDL	High-density lipoprotein cholesterol
HOMA-IR	Homeostatic Model Assessment of Insulin Resistance
LDL	Low-density lipoprotein cholesterol
LH	Luteinizing hormone
FSH	Follicle-stimulating hormone
LH/FSH	Luteinizing hormone-to-follicle-stimulating hormone ratio
OR	Odds ratio
PCOS	Polycystic ovary syndrome
PMOS	Polyendocrine metabolic ovarian syndrome
TPOAb	Thyroid peroxidase antibodies
TSH	Thyroid-stimulating hormone
