# Supplementary material for: Thyroid Autoimmunity in Polycystic Ovary Syndrome: Phenotype Distribution, HDL-Cholesterol, and Data-Driven Clusters in a Retrospective Cohort Study
Source: Medicina (Kaunas). 2026 Jun 18;62(6):1184. doi: 10.3390/medicina62061184 (PMC13304434; doi:10.3390/medicina62061184)
Supplement: Supplementary file 1 [file medicina-62-01184-s001.zip › Supplementary Table S1 pmos.pdf]

Supplementary Table S1. BMI, thyroid-related parameters, and treatment characteristics according to autoimmune thyroiditis status in women with PCOS

Variable	No AIT	AIT	p-value
**BMI, kg/m²**	28.5 (24.8–33.0)	31.0 (24.0–36.0)	0.562
Available data, n	86	101	
**Anti-TPO antibody titer**	10.9 (10.0–18.2)	84.9 (28.4–810.0)	0.004
Available data, n	15	20	
**Anti-thyroglobulin antibody titer**	20.0 (20.0–202.5)	126.5 (58.6–210.5)	0.243
Available data, n	10	9	
**Thyroid functional status**			0.008
Euthyroid	36 (81.8%)	48 (92.3%)	
Subclinical hyperthyroidism	4 (9.1%)	0 (0%)	
Subclinical hypothyroidism	4 (9.1%)	1 (1.9%)	
Overt hypothyroidism	0 (0%)	3 (5.8%)	
Available data, n	44	52	
**Treatment characteristics**			
Levothyroxine	20 (16.4%)	38 (26.8%)	0.052
Antithyroid drugs	29 (23.8%)	11 (7.7%)	<0.001
Beta-blockers	8 (6.6%)	5 (3.5%)	0.271
Metformin	10 (8.2%)	4 (2.8%)	0.059
Oral contraceptives	47 (38.5%)	68 (47.9%)	0.137

Data are presented as median (Q1–Q3) or n (%).
AIT = autoimmune thyroiditis; BMI = body mass index; anti-TPO = anti-thyroid peroxidase antibodies.
