# Supplementary material for: Thyroid Autoimmunity in Polycystic Ovary Syndrome: Phenotype Distribution, HDL-Cholesterol, and Data-Driven Clusters in a Retrospective Cohort Study
Source: Medicina (Kaunas). 2026 Jun 18;62(6):1184. doi: 10.3390/medicina62061184 (PMC13304434; doi:10.3390/medicina62061184)
Supplement: Supplementary file 1 [file medicina-62-01184-s001.zip › Supplementary Table S2 pmos.pdf]

Supplementary Table S2. Multivariable sensitivity analyses evaluating the association between autoimmune thyroiditis and lipid parameters after adjustment for BMI, cardiometabolic comorbidities, and treatment exposure

**Predictor**	**Total cholesterol β (SE)**	**p-value**	**HDL-cholesterol β (SE)**	**p-value**	**LDL-cholesterol β (SE)**	**p-value**
Autoimmune thyroiditis	5.53 (6.64)	0.407	−1.74 (3.62)	0.633	8.63 (10.82)	0.428
BMI	−0.13 (0.48)	0.788	−0.43 (0.24)	0.078	0.65 (0.73)	0.376
Diabetes mellitus	−3.14 (8.88)	0.724	−5.77 (4.87)	0.241	4.98 (14.62)	0.734
Hypertension	5.74 (8.36)	0.493	−3.40 (4.42)	0.445	5.28 (12.31)	0.669
Levothyroxine treatment	8.03 (7.70)	0.299	−2.31 (3.86)	0.551	−3.72 (11.21)	0.741
Metformin treatment	5.94 (14.15)	0.675	−0.41 (6.76)	0.952	−8.38 (19.50)	0.669
Oral contraceptive use	10.50 (6.30)	0.097	−2.28 (3.49)	0.516	9.27 (10.40)	0.376

All models were additionally adjusted for age using restricted cubic splines (3 degrees of freedom).
AIT = autoimmune thyroiditis; BMI = body mass index; β = regression coefficient; SE = standard error.

Model characteristics:

- Total cholesterol model: adjusted $R^2 = 0.110$; overall model $p = 0.001$.
- HDL-cholesterol model: adjusted $R^2 = 0.055$; overall model $p = 0.162$.
- LDL-cholesterol model: adjusted $R^2 \approx 0.000$; overall model $p = 0.453$.
